# Insights from Binding on Quadruplex Selective Carbazole Ligands

**DOI:** 10.1002/chem.202101866

**Published:** 2021-07-19

**Authors:** Diana Müller, Puja Saha, Deepanjan Panda, Jyotirmayee Dash, Harald Schwalbe

**Affiliations:** ^1^ Institute of Organic Chemistry and Chemical Biology Center for Biomolecular Magnetic Resonance (BMRZ) Goethe University Frankfurt Max-von-Laue Strasse 7 Frankfurt am Main 60438 Germany; ^2^ School of Chemical Sciences Indian Association for the Cultivation of Science Jadavpur Kolkata-700032 India

**Keywords:** biophysics, carbazole ligands, G-quadruplexes, ligand design, NMR spectroscopy

## Abstract

Polymorphic G‐quadruplex (G4) secondary DNA structures have received increasing attention in medicinal chemistry owing to their key involvement in the regulation of the maintenance of genomic stability, telomere length homeostasis and transcription of important proto‐oncogenes. Different classes of G4 ligands have been developed for the potential treatment of several human diseases. Among them, the carbazole scaffold with appropriate side chain appendages has attracted much interest for designing G4 ligands. Because of its large and rigid π‐conjugation system and ease of functionalization at three different positions, a variety of carbazole derivatives have been synthesized from various natural or synthetic sources for potential applications in G4‐based therapeutics and biosensors. Herein, we provide an updated close‐up of the literatures on carbazole‐based G4 ligands with particular focus given on their detailed binding insights studied by NMR spectroscopy. The structure‐activity relationships and the opportunities and challenges of their potential applications as biosensors and therapeutics are also discussed. This review will provide an overall picture of carbazole ligands with remarkable G4 topological preference, fluorescence properties and significant bioactivity; portraying carbazole as a very promising scaffold for assembling G4 ligands with a range of novel functional applications.

## Graphical abstract

**A summary** is provided of the most promising carbazole ligands that have been established as effective G‐quadruplex binders. The focus is made on the structural design of carbazole ligands and on NMR analysis of carbazole‐G4 interactions with particular attention to the variety in binding modes shown by the carbazole ligands. Further, a brief view on biophysical and biological data of their molecular interactions with G‐quadruplexes has been discussed.

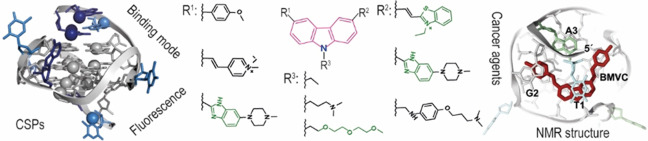

## 1 Introduction

**Figure 1:**
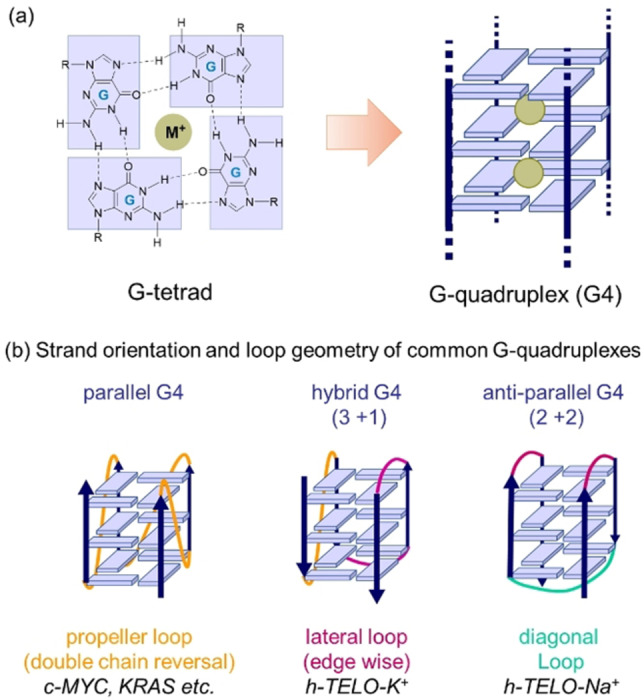
(a) G‐quadruplex (G4) formation via stacking of Hoogsteen hydrogen bonded G‐tetrads. (b) Structure and polymorphism of major type of G4s.

G‐quadruplexes (G4s) are considered as promising drug targets for therapeutic applications.^[1]^ The four‐stranded G‐quadruplex structures are basically formed by guanine‐rich DNA or RNA sequences in near‐promoter, telomere or UTR regions.^[2]^ Quadruplexes play pivotal roles in the regulation of replication, transcription and translation as well as in the maintenance of telomere length homeostasis.[^2^, ^3^] Given the significance of quadruplexes in biological functions, much effort has been put into the development of effective G4 binders derived either from natural products or synthetic compounds.^[4]^ G4s are composed of three or more layers of stacked G‐tetrads that are formed by four guanine residues through Hoogsteen hydrogen bonds^[5]^ (Figure 1a). The square planar alignment of G‐tetrads provides a unique site for specific recognition of quadruplexes via π‐π stacking interactions. G4 binding can also be controlled by interaction with G4 loops or grooves (Figure 1b) via electrostatic interactions or intercalation between G‐tetrads or by a combination of all these modes.

**Figure 2:**
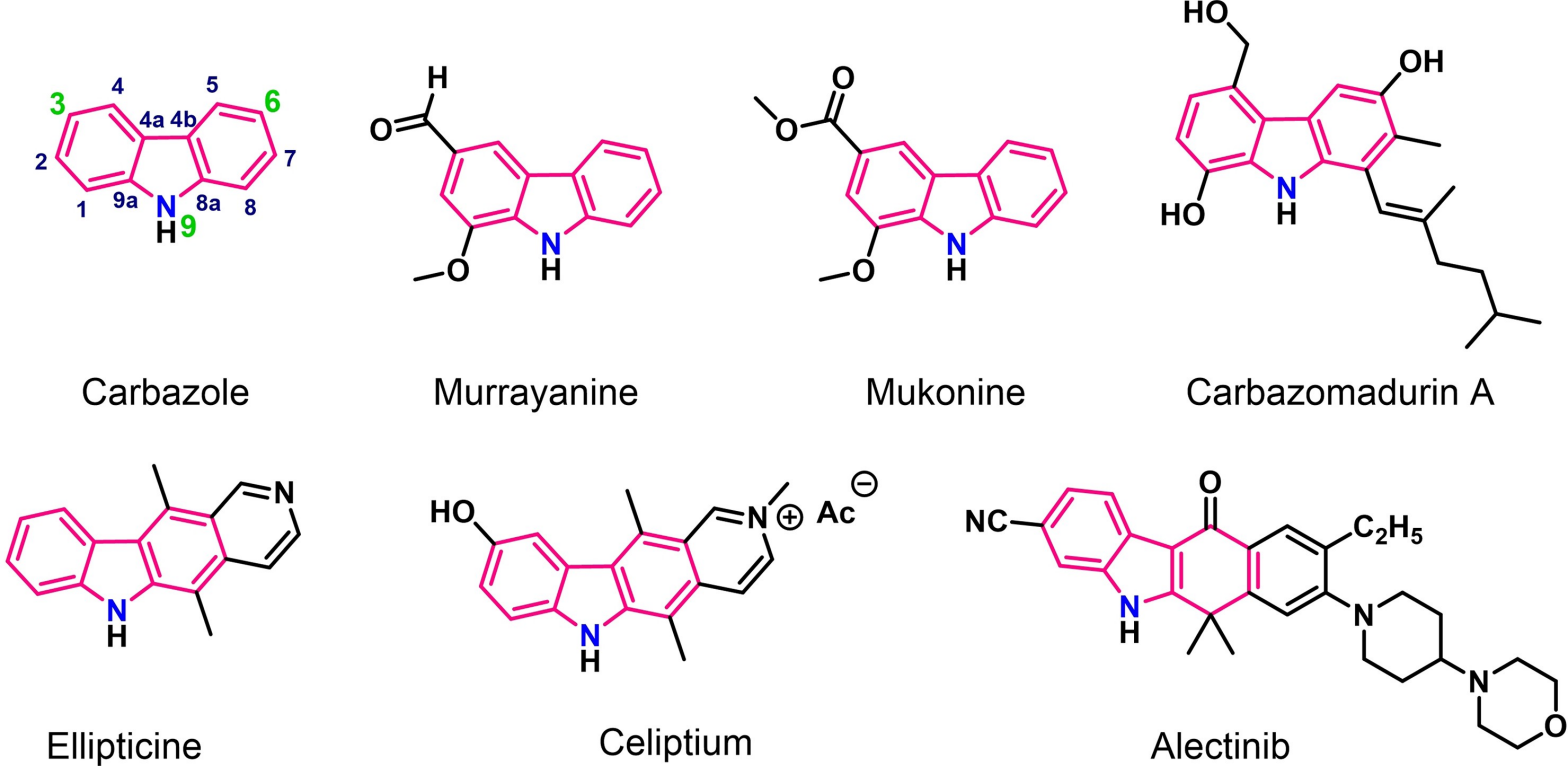
Chemical structures of carbazole and its derivatives that show potent pharmacological activities such as anti‐inflammatory, anti‐microbial (Murrayanine),^[8b]^ antiproliferative (Mukonine),^[9b]^ antioxidant (Carbazomadurin A),^[11]^ and antitumor (Ellipticine,^[14]^ Celiptium,^[17]^ Alectinib^[17]^).

Given the unique feature of quadruplex recognition through G‐tetrads, most of the selective G4 binders are those that possess a large flat‐shaped aromatic surface that is much larger than that of a duplex binder to avoid non‐specific interactions with DNA duplexes.^[3]^ Heteroaromatic scaffolds like carbazoles have been designed as potential quadruplex binding ligands. Carbazole, a strong pharmacophoric moiety, is a tricyclic structure consisting of two benzene rings fused on both sides of a nitrogen‐containing five‐membered ring (Figure 2). This heterocyclic ring system can stack upon the top or bottom of external G‐tetrads of quadruplexes by π–π interactions. The core system of a carbazole can also be linked with one‐to‐three external side chains like cationic arms to exploit electrostatic interactions with negatively charged phosphate backbones or with some side‐chain appendages for hydrogen‐bonding interactions with the G4 groove/loop regions. Of important note, carbazole derivatives with appropriate functional groups can adopt crescent shaped topologies enabling a well‐defined interaction with intramolecular G‐quadruplexes. The structural unit is also able to display spectroscopic properties that can further provide potential means for monitoring binding interactions with quadruplexes. Based on these intriguing features, carbazoles have attracted considerable interest for targeting various G‐quadruplex structures.

Not surprisingly, the carbazole ring system has thus been a key chemical scaffold in a variety of biologically active compounds.^[6]^ Owing to a wide spectrum of bioactivity and therapeutic properties, carbazoles have been considered as a potential drug candidate for the treatment of multiple diseases like cancer, diabetes, viral and bacterial diseases, neurological disorders etc.^[7]^ The carbazole ring is also prevalent in several medicinally active natural products for example, murrayanine,^[8]^ mukonine,^[9]^ carbazomycins,^[10]^ carbazomadurin A,^[11]^ staurosporine,^[12]^ murrayafoline A^[13]^ (Figure 2). In addition, many carbazole derivatives have been chemically synthesized and are well established for their pharmacological activities. More interestingly, there are numerous carbazole based commercially drugs that are available in the market such as ellipticine,^[14]^ olivacine,^[15]^ datelliptium,^[16]^ alectinib,^[17]^ celiptium^[17]^ etc (Figure 2).

With the present article, we intend to provide a brief, up‐to‐date summary of the most promising carbazole based ligands that have been established as effective G‐quadruplex binders. Small molecules targeting G4 have been reviewed in many literatures with different perspectives. Here, the aim of this article is to delineate structural design of carbazole ligands and the essential biophysical and biological data for understanding their molecular interactions with G‐quadruplexes and particular notion has been given on in‐depth NMR analysis of carbazole‐G4 interactions.

## 2 Binding Mode of Quadruplex‐interactive Carbazole Ligands

Nuclear magnetic resonance (NMR) spectroscopy is a powerful method for understanding G‐quadruplex structures as well as their interaction with ligands of low molecular weight (“small ligands”).^[18]^ It allows insight into the binding of quadruplex‐interactive ligands at atomic level under near‐physiological conditions. Beside structural information, information about dynamics and kinetics can be obtained. For the investigation of ligand‐G4 interactions, it is a substantial advantage that G4 signals deriving from Hoogsteen base pairs are well separated from the signals of Watson–Crick or i‐motif base pairs.^[19]^ Investigation of the 1D NMR spectral region featuring imino signals allows a fast check for the homogeneity of the G4 folding simply by counting the number of NMR signals.[^5g^, ^20^] Further, from initial 1D ^1^H NMR titration experiments, information regarding the exchange regime (k_ex_) of ligand binding can be gained. It is generally classified into three regimes on NMR time scales: fast (k_ex_≫Δδ; Δδ=difference of chemical exchange between apo and holo state), intermediate (k_ex_≈Δδ) and slow exchange (k_ex_≪Δδ). Ligand binding in fast exchange leads to a shift of the signals (also referred to as chemical shift perturbation (CSP)) whereas binding in intermediate exchange results in line broadening (up to beyond detection limit). In slow exchange, a new set of signals from the G4‐ligand complex appears. Under these conditions, together with further experiments, the determination of a high‐resolution structure could be feasible.^[18]^ The scarce number of solution NMR‐structures of DNA G4‐ligand complexes^[21]^ is caused by the scarce number of ligands that bind in slow exchange. However, G4 structures in the ligand‐free state are used for rational structure‐based drug design. Signals that experience the strongest effect of intermediate or fast exchange binding ligands can be depicted on the respective G4‐structure or docking studies can be performed to estimate the binding site.^[22]^ Interpretation of such docking studies have to be conducted with caution as the capping structure of the free G4 may not reflect the conformation in the ligand‐bound state.^[21e]^

**Figure 3:**
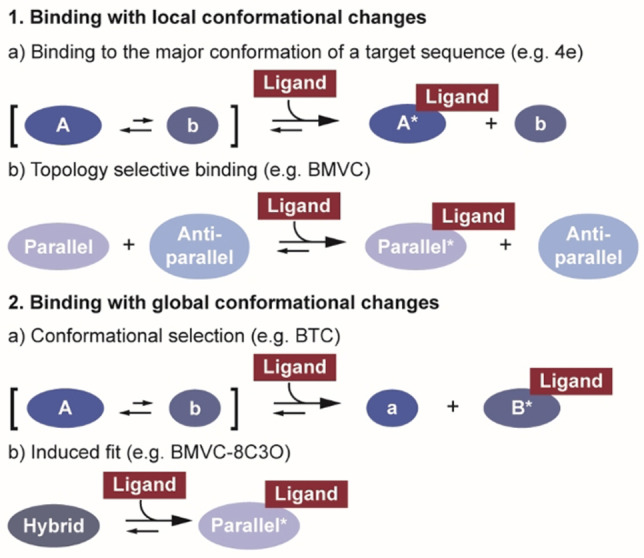
Schematic drawing of binding modes found for carbazole ligands. They have been divided into two main categories: Binding with local and binding with global conformational changes. The first category includes ligands that induce changes around the binding pocket and they were either designed to bind to the major conformation (A) of the target sequence or to target solely in a topology selective manner. Whereas in the conformational selection mechanism, the ligand binds to a pre‐existing minor conformation (b) and influences the conformational equilibrium in favour to the binding competent form. Further, the ligand can induce a change in the overall topology of the binding partner and follow an induced fit mechanism.

As most carbazole ligands have been found to bind in intermediate exchange regime, they will be classified according to their binding mode (Figure 3) in the further sections. The different binding modes found for G4 DNA targeting carbazole ligands have been divided into two main sections: Binding with local or with global conformational changes (Figure 3). Binding with local changes has been further differentiated by the purpose of ligand design and refers to ligand binding to the major conformation of a targeted sequence (Figure 3a) and binding to a specific G4 topology irrespective of the sequence (Figure 3b). Global conformational changes can either be induced by a conformational selection or induced fit mechanism.^[23]^ It is often difficult to differentiate whether the conformational change takes place before (conformational selection) or after (induced fit) ligand binding. In order to determine the mechanism, all rate constants at varying substrate:ligand concentrations would have to be known.^[23]^ Even though NMR is the best technique to evaluate these parameters, the binding modes of the here presented ligands have not been examined in detail. Often, the ligand solubility is insufficient to reach the required excess of ligand. Hence, the classification into conformational selection occurred on the basis of a pre‐existing minor conformation in slow exchange that has been increasingly populated upon ligand addition.

### 2.1 Binding to the major conformation of a target sequence

**Figure 4:**
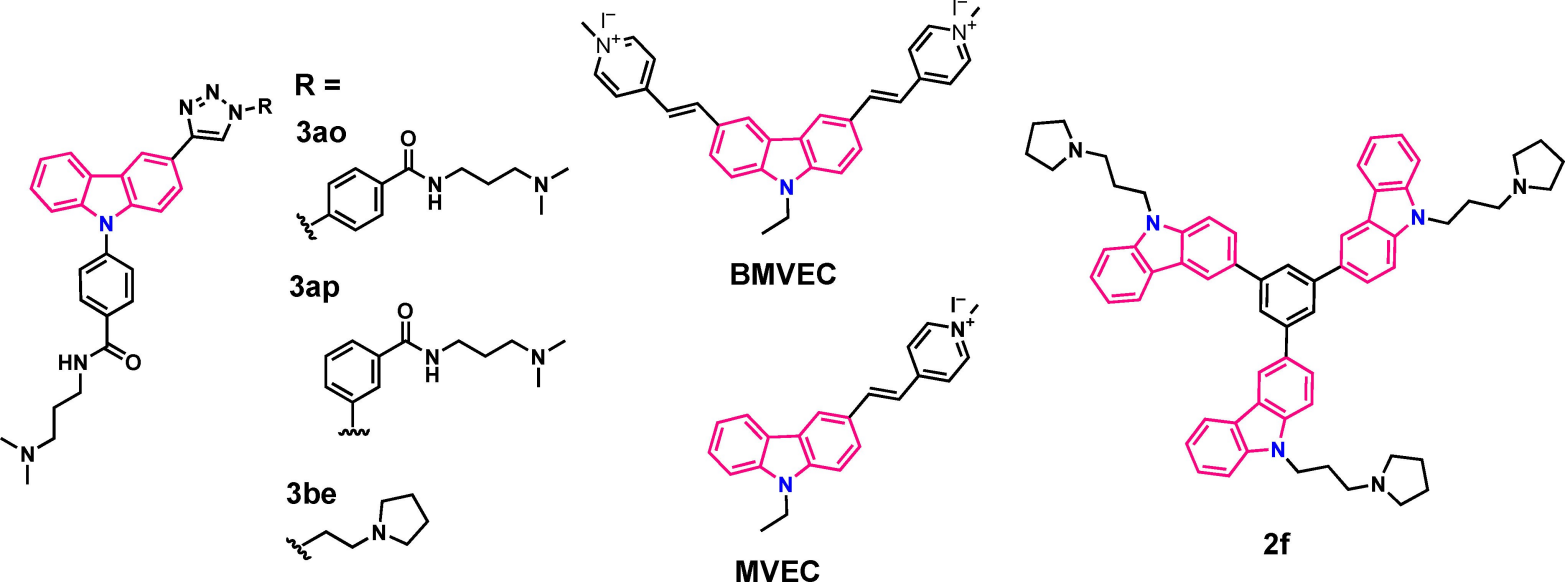
Chemical structures of carbazole ligands capable of binding to the major conformation of G4 DNA.

Many carbazole ligands bind to the major conformation of the G4 target sequence without a recognizable effect on the conformational equilibrium or the overall topology. The carbazole ligands **3ao**,^[24]^ **3ap**,^[24b]^ **BMVEC** ^[25]^ and **2f** ^[26]^ (Figure 4) can be categorized within this group. Ligand **3ao** (alternatively known as **Tz1**) and **3ap** are mono‐triazolyl carbazole derivatives that have been developed by in situ copper free click reaction in the presence of *c‐MYC* G4^.^Au@Fe_3_O_4_ and *BCL*2 G4^.^Au@Fe_3_O_4_ nanotemplates.^[24]^ **3ao** containing two aryl carboxamide motifs is capable of specifically recognizing *c‐MYC* parallel G‐quadruplex (K_d_=170 nM) over i‐motifs and *BCL2* G4 while the meta‐isomer **3ap** prefers to bind to *BCL2* G4 DNA with a dissociation constant in comparatively high micro molar regime (K_d_=0.68 μM). In case of **3ao**, significant line broadening of the imino signals has been observed at a ratio of [**3ao**]:[*c‐MYC*]=0.25 indicating strong binding interactions. These molecules have been shown to reduce off‐target effects in biological experiments; ligand **3ao** and **3ap** could selectively downregulate the expression of *c‐MYC* and *BCL2* genes, respectively via stabilization of promoter quadruplexes and eventually lead to apoptosis in cancer cells. Another carbazole ligand **3be** ^[24b]^ generated from in situ cycloaddition with *c‐MYC* i‐motif DNA nanotemplate (*c‐MYC* C4^.^Au@Fe_3_O_4_) results in general line broadening of the characteristic imino signals (15–16 ppm) for i‐motif C–C^+^ base pairs as well as in the aromatic region of *c‐MYC* i‐motif.

Tang and coworkers reported two carbazole iodides **MVEC** [(3‐Bis‐(1‐methyl‐4‐vinylpyridium iodine) 9‐ethyl‐carbazole)] and **BMVEC** [(3,6‐Bis‐(1‐methyl‐4‐vinylpyridium iodine) 9‐ethyl‐ carbazole)] to explore the binding mode of carbazole derivatives with telomeric G4 DNA (Figure 4).^[25]^ **BMVEC** has cationic charge on the two pendant groups of pyridinium rings of 9‐ethylcarbazole while **MVEC** has one cationic charge pendant group. CD spectroscopy showed that **BMVEC** significantly stabilizes both parallel *Hum6* (T_2_AG_3_) (Δ$T_{m}^{CD}$ =16 °C at ligand:G4=1 : 1) and antiparallel *Hum22* (AT_2_G_3_T_2_AG_3_) telomeric G4 DNA (Δ$T_{m}^{CD}$ =7 °C at ligand:G4=1 : 1) but **MVEC** prefers to bind to parallel *Hum6* DNA (Δ$T_{m}^{CD}$ =10 °C at ligand:G4=1 : 1). An induced CD‐signal was observed near 300 nm on binding of **BMVEC** to basket‐type antiparallel *Hum22*. 1D ^1^H NMR spectroscopy further revealed that **BMVEC** interacts with *Hum6* G4 DNA in slow exchange on NMR time scale. The imino proton signals (10–12 ppm) of *Hum6* G4‐G6 gradually disappeared and a new set of imino proton resonances appeared at a 1 : 1 **BMVEC**:*Hum6* stoichiometry. The authors conclude that at low concentration, **BMVEC** initially binds to the groove region and at higher concentrations, it then end stacks onto the G‐tetrad near the G6 residue. NMR titrations with **MVEC** result in progressive upfield shifts of G4‐G6 signals and line broadening of G4 signal at **MVEC**:*Hum6* ratio of 1 : 1 indicating significant interaction with the *Hum6* G4 residue in an intermediate exchange regime. In case of *Hum22*, **BMVEC** induces only significant line broadening of the imino resonance signals (10.5–12.0 ppm). Both these carbazole iodides were shown to exhibit anti‐proliferative activity against hepatocellular and colorectal carcinoma cell lines.

A novel multi‐carbazole derivative consisting of a tri(carbazole)benzene core was identified as a promising G4‐stabilizer by Mergny and Smith group.^[26]^ They envisaged that the hetero‐polyaromatic scaffold with C3‐symmetry and a crescent shaped architecture could enable effective stabilization by *π*‐stacking interactions and the three protonated N‐alkylamine side chains angled in appropriate positions on the scaffold could facilitate selective interactions with loop and groove regions of G‐quadruplexes (Figure 4). The multi‐carbazole ligand **2f** with C3‐alkyl chain length and pyrrolidine cyclic amine substituents exhibited a high degree of stabilization for hybrid and antiparallel 21‐mer telomeric G4 DNA in DNA‐based FRET melting studies (ΔT_m_=14.3 °C; ligand conc. 5 μM, ligand:G4=25 : 1) and for parallel *KRAS* G4 DNA (ΔT_m_=22.7 °C; at 5 μM ligand concentration). 1D ^1^H NMR spectroscopy with *KRAS* G4 demonstrated severe line broadening and decreased intensity of all the imino signals (10–12 ppm) of the guanine residues involved in the formation of G‐quartets.

**Figure 5:**
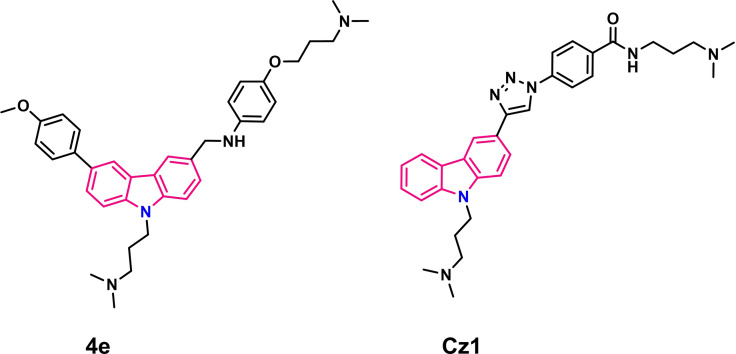
Chemical structure of carbazole **4e** and **Cz1**.

For the ligands **4e** ^[27]^ and **Cz1**,^[28]^ a more specific interaction with *c‐MYC22* could be observed (Figure 5). *c‐MYC22* is a stabilized mutant of the purine‐rich nuclease hypersensitive element NHEIII_1_ located upstream of the *c‐MYC* promoter sequence.^[29]^

**Figure 6:**
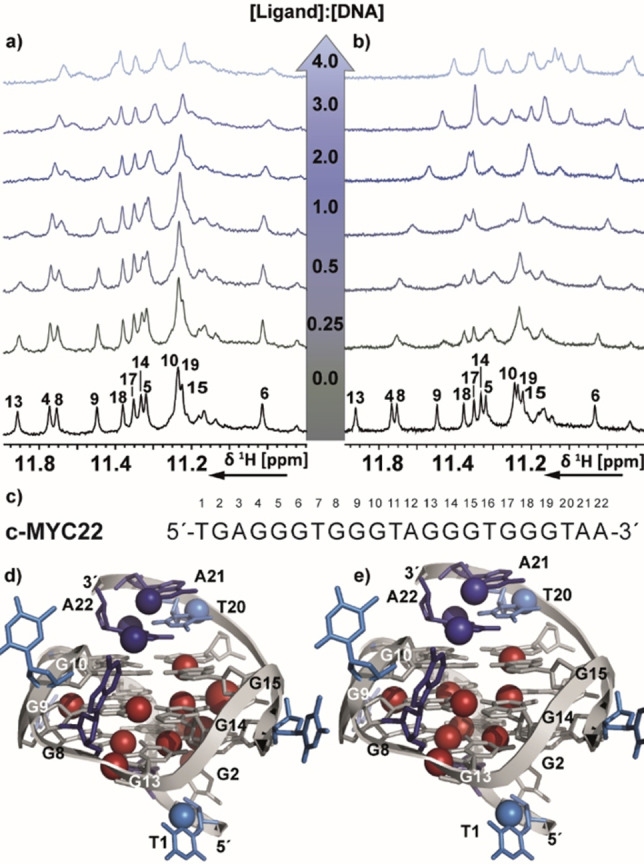
Imino region of 1D ^1^H NMR spectrum of the *c‐MYC22* G‐quadruplex DNA with increasing [ligand]:[DNA] molar ratio of (a) **4e** and (b) **Cz1**. b) The spectra were recorded at 298 K, 600 MHz. Experimental conditions: 100 μM DNA in 25 mM Tris ⋅ HCl (pH 7.4) buffer containing 100 mM KCl in 5 % d^6^‐DMSO/95 % H_2_O. c) sequence of *c‐MYC22* with the numbering used for assignment that has been transferred from *Ambrus* et al..^[29]^ d) and e) Mapping of the observed changes in 1D ^1^H NMR spectra upon addition of **4e** and **Cz1**, respectively, on the solution NMR structure of *c‐MYC*22 (PDB:1XAV). T, A and G are light blue, dark blue and grey, respectively. GH1 and GH8, TH6, AH8 and AH2 that experience chemical shift perturbation are highlighted with red, light blue and dark blue spheres, respectively. The mapping reveals that both ligands affect similar signals, which are located at the external tetrads and the groove formed by the G‐stretches 8–9‐10 and 13–14‐15. Therefore, an identic binding site for both ligands is expected. However, **Cz1** shows stronger binding as compared to **4e**.

Carbazole ligand **4e** ^[27]^ was developed by Dash group via dynamic combinatorial approach (DCC) from an imine‐based combinatorial library in the presence of *c‐MYC* G4 DNA (NMR data not published). Both these ligands exhibited high selectivity for quadruplex over duplex DNA. The ligands showed a clear binding preference for *c‐MYC* G4 [**4e**: ΔT_m_=23.4 °C (ligand concentration 1 μM, ligand:G4=5 : 1), K_d_=1.08 μM; **Cz1**: ΔT_m_=15.8 °C (ligand concentration 1 μM, ligand:G4=5 : 1), K_d_=0.21 μM] and acted as endogenous transcriptional regulators of *c‐MYC* gene in cancer cells. These compounds were subsequently shown to induce cytotoxicity and apoptosis in cancer cell lines. In case of **4e**, the imino signals of G8, G9, G13 and G14 were more affected as well as one or two signals in the overlapping region of G10, G15 and G19 (Figure 6a) (numbering according to Figure 6c).

In the aromatic region, line broadening and CSPs were also observed for signals of the flanking nucleotides T1, G2, T20, A21 and A22 (data not shown). In molecular docking studies, binding to the 3'‐external tetrad was observed. The NMR data provide supporting evidence that both external tetrads act as potential binding sites as well as interaction of **4 e** with the groove formed by G8‐G9‐G10 and G13‐G14‐G15. Titration of **Cz1** resulted in strong line broadening of the imino protons of *c‐MYC22* (NMR data not published). However, upon a 1 : 1 molar ratio of **Cz1**:*c‐MYC22*, the binding was shifted into fast exchange resulting in CSPs and an increase in signal intensity (Figure 6b). Altogether, most affected signals were also located at the groove formed by the G‐stretches 8–9‐10 and 13–14‐15 and the aromatic signals of T1, G4, T20 and A22 (data not shown). Thus, a similar binding site for **Cz1** like **4 e** is expected (Figure 6d–e).

**Figure 7:**
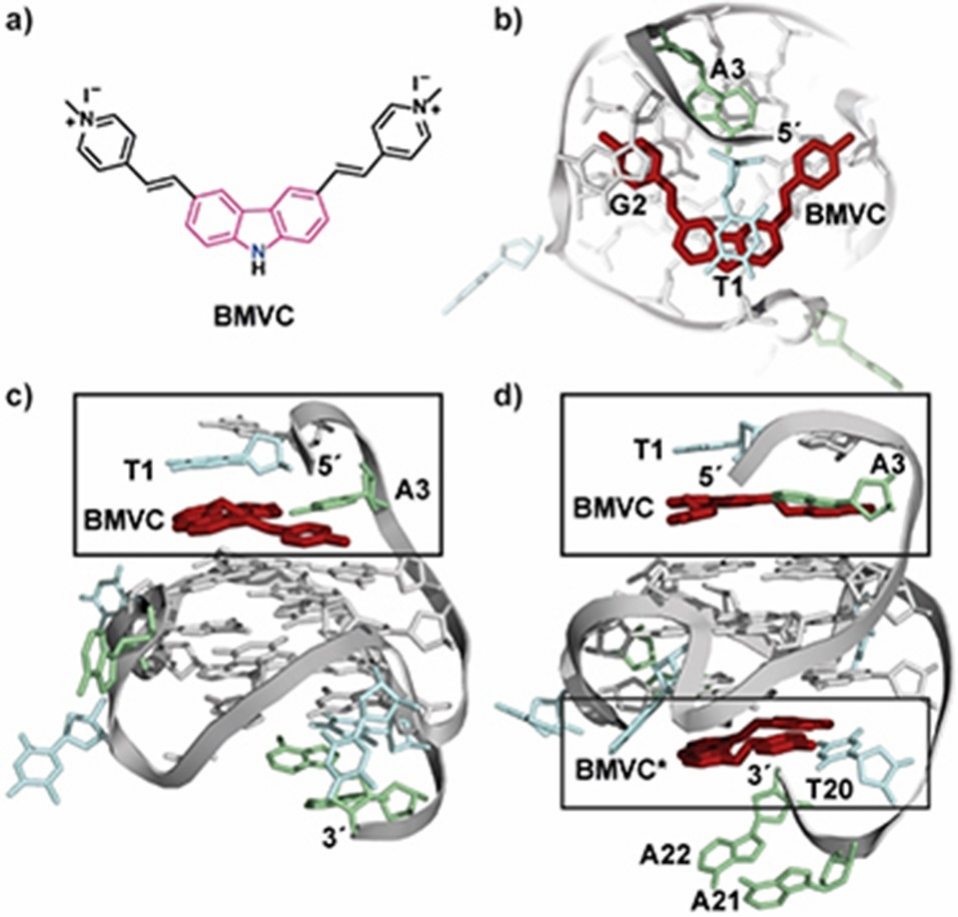
(a) Chemical structure of ligand BMVC. (b) top view onto the 5’‐end of the 1 : 1 BMVC:*c‐MYC22* complex (PDB: 6JJ0).^[30]^ (c) side view of the 1 : 1 complex and (d) side view of the 2 : 1 BMVC : *c‐MYC22* complex (PDB: 6O2L),^[30]^ BMVC* indicates the weaker binding site. A‐, T‐ and G‐residues are depicted in green, blue and grey, respectively.

**BMVC** (3,6‐bis(1‐methyl4‐vinylpyridium)carbazole diiodide) is one of the few ligands that binds in slow exchange rate to *c‐MYC22* and the NMR solution structures of the 1 : 1 (PDB code 6JJ0) and 2 : 1 (PDB code 6O2L) complex were reported^[30]^ (Figure 7, numbering according to Figure 6c). The crescent shaped ligand is highly sensitive and efficient light‐up fluorescent probe for G4 DNA due to its good water solubility and biocompatibility. NMR studies revealed that the ligand first binds to the 5'‐end and forms a tight complex. Moreover, at a ligand:DNA molar ratio of 1 : 1, signals of the 2 : 1 complex are also coming up with the 3' external tetrad as additional binding site. Interestingly, the 5’ (T4, G5 and A6) and 3’ flanking segments underwent large conformational changes creating additional planes that serve as potential binding sites for **BMVC. BMVC** also adopts a contracted conformation that allows optimal π‐π stacking with both the external tetrads and 5’‐ and 3’ capping structures. 2D NMR analysis provided detailed binding mode of **BMVC**: The G2 and A3 residues at the 5’‐capping segment stack over G4 of the 5’‐tetrad while A3 inserts into the arc of **BMVC** forming a **BMVC**‐A3 plane. The T1 stacks on the central carbazole core of **BMVC** further stabilizing the **BMVC**‐A3 plane. At the 3‘‐end, T20 forms a new plane with **BMVC** and stack upon 3’‐tetrad. However, the 3'‐binding site showed a weaker affinity (as the A21 and A22 residues do not participate in stacking interactions with **BMVC**‐T20 plane) and displayed a high sensitivity to T20A mutation or truncation of the TAA‐flanking sequence. These indicate that **BMVC** binds the *MYC* G4 with greater affinity (K_d_=36 nM) and specificity, considerably stronger than other reported G4 ligands and represses *c‐MYC* gene expression in cancer cells.

### 2.2 Topology selective ligand

**Figure 8:**
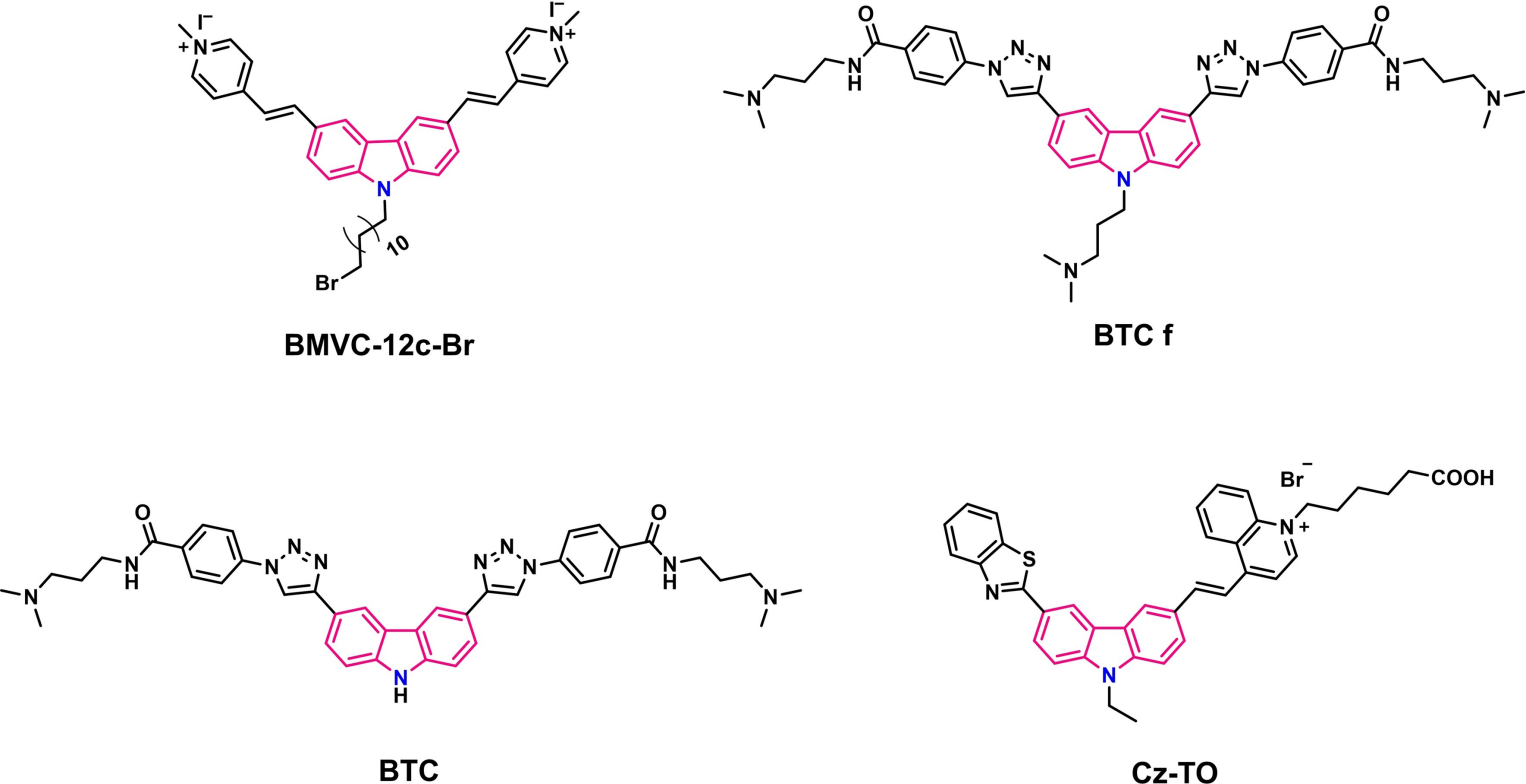
Structures of topology selective ligand **BMVC‐1c‐Br** and specific G4‐conformation selective carbazole derivatives **BTC**, **BTC f** and carbazole‐thiazole orange conjugate **Cz**‐**TO**.

Beside ligands that bind specifically to a particular promoter G4 there are also topology‐specific ligands. The group of Ta‐Chau Chang synthesized a derivative of **BMVC** with a high selectivity towards parallel G4s: **BMVC‐12C**‐**Br** (Figure 8).^[31]^ Owing to the presence of dodecyl alkyl chain, the derivative acts as a surfactant between oil and water. Based on this hydrophobicity, they developed a method to separate G4 of different topologies ‐ the emulsified induced filtration (EIF). A two‐phase system containing a mixture of G4s in the aqueous phase and the ligand as surfactant between the phases was used. As model systems, a mixture of the hybrid‐type Tel24‐M^[32]^ and predominantly parallel Tel19‐M^[33]^ telomeric sequences as well as a mixture of the two promoter sequences *c‐MYC*‐2345 (parallel)^[34]^ and *BCL2*mid‐M (hybrid)^[35]^ were used. By ultrasonic emulsification, an oil‐in‐water emulsion has been formed and filtrated through a membrane of mixed cellulose esters (MCEs). With this process, a transfer of the parallel G4 into the emulsion particles by binding to **BMVC‐12C**‐**Br** resulting in a structural separation was nicely reported by NMR.^[31]^

### 2.3 Conformational selection

**Figure 9:**
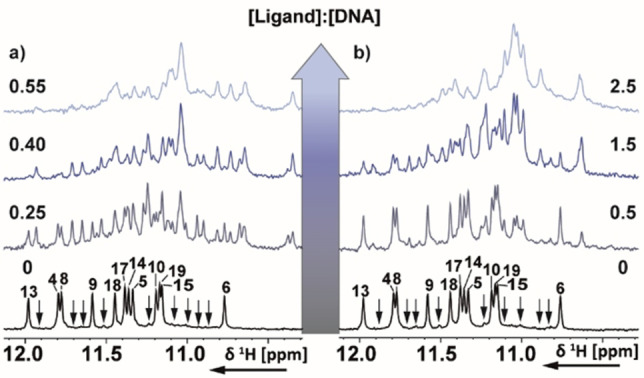
Imino region of 1D ^1^H NMR spectrum of the *c‐MYC22* G‐quadruplex DNA with increasing [Ligand]:[DNA] molar ratio of (a) **BTC** and (b) **BTC f**. The spectra were recorded at 298 K, 600 MHz. Experimental conditions: 100 μM DNA in 25 mM Tris ⋅ HCl (pH 7.4) buffer containing 100 mM KCl in 10 % D_2_O/90 % H_2_O. Both ligands bind to a pre‐existing minor conformation and induce a change in conformational equilibrium of *c‐MYC22*. The numbering is according to Figure 6c). a) is adapted with permission from Ref. [36]. Published by The Royal Society of Chemistry. b) is adapted with permission from Ref. [37]. Copyright 2015, Springer Nature.

In the conformational selection binding mode, the ligand interacts to a pre‐existing conformation of a biomolecule, only present in small amounts and shifts the conformational equilibrium towards the binding competent form.^[23]^ The carbazole ligands **BTC** ^[36]^ and **BTC‐f** ^[37]^ (Figure 8), developed by Dash group, select the minor‐populated conformer (in Figure 3 designated as **b**) of *c‐MYC* G4 DNA and binds to it in a specific manner. Both these bis‐triazolyl carbazole derivatives were prepared by one‐pot Cu(I) catalysed Huisgen 1,3‐dipolar azide‐alkyne cycloaddition. **BTC f** with a *N*‐alkylated NMe_2_ side chain in carbazole moiety showed a ΔT_m_ value of 22.7 K in FRET based DNA melting studies at only 100 nM ligand concentration (ligand:G4=1 : 2) and a K_d_ of 300 nM and ligand **BTC** lacking the central NMe_2_ side chain displayed quite similar binding affinity for *c‐MYC* (ΔT_m_ value of 24 K at 200 nM ligand concentration, ligand:G4=1 : 1 and a K_d_ of 450 nM). The ligands were titrated to *c‐MYC22* G4 DNA, a stabilized mutant of wild‐type *c‐MYC* Pu27 that presents additional signals presumably belonging to multiple structures of the 3'‐capping formed by the TAA‐flanking nucleotides. Both the ligands were found to induce an increase in signal intensity of the minor conformation (**b**) of *c‐MYC22* while reducing the signals of the major conformation (**A**) (Figure 3 and Figure 9). Moreover, upon ligand addition, new signals appeared in the imino region (e.g., at 10.5 ppm) suggesting that the binding‐competent minor conformation (**b**) underwent small conformational changes (B*) (like restructuring of the capping structure) in the presence of **BTC** and **BTC‐f**. Interestingly, these ligands were found to stabilize the *c‐MYC* minor conformation formation even in the absence of any added K^+^ ion.

A fluorescent G4‐binding dye ‐ the carbazole‐thiazole orange conjugate **Cz**−**TO** (Figure 8) was found to bind the *BCL2* 2345 G4 DNA via end‐stacking as the signals of tetrad‐forming guanines are more affected by binding.^[38]^ This observation is not comprehensible for us. However, there are some signals (approximately at 11.05, 11.3 and 11.45 ppm) increasing in intensity upon ligand addition to *BCL2* 2345 that were ignored by the authors. Considering the spectral changes resulted by **BTC** and **BTC f** binding, a conformational selection mechanism seems plausible. As only 2 datapoints (0 eq and 0.75 eq) were published, a detailed analysis is not possible. However, **Cz**‐**TO** specifically enhances its fluorescence intensity over 70‐fold in the presence of *BCL2* G4 while showing less than 30‐fold increase with promoter (*c‐MYC*, *c‐KIT1* and *VEGF*) and telomeric G4s and other nucleic acids.

### 2.4 Induced fit

**Figure 10:**
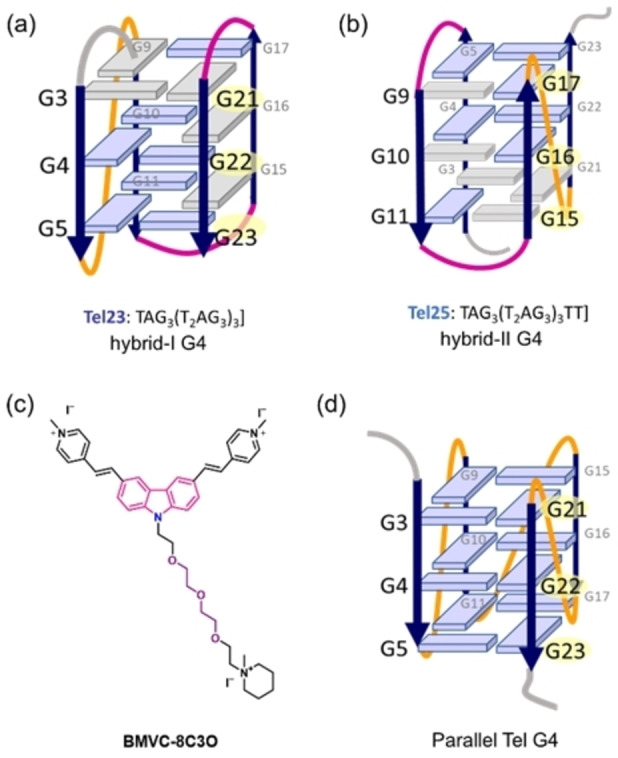
Induced‐fit binding mechanism of G4‐interactive ligand. Ligand **BMVC‐8C3O** induces conformational transition from (a) hybrid‐I and (b) hybrid‐II telomeric G4 topology to (d) parallel topology.

Ligand **BMVC‐8C3O** was shown to convert the overall topology of its G4 binding partner (Figure 10).^[39]^ This is typical for an induced fit binding mode.^[23]^ The ligand was prepared by Chang and co‐workers via covalent attachment of a tetraethylene glycol (8C3O) terminating in a methyl‐piperidinium cation with the carbazole diiodide derivative **BMVC**. Upon interaction with **BMVC‐8C3O**, the hybrid type Tel23 (hybrid I) (Figure 10a) and Tel25 (hybrid II) (Figure 10b) G4s topologically convert from their apo form to a parallel structure (Figure 10d).

The authors recorded NMR spectra in 99 % D_2_O at several time points (10 min to 24 h) upon ligand addition. In D_2_O, only the imino protons of the inner tetrad that are protected for exchange are visible. Thus, the transformation from the hybrid to the parallel structure over time can be easily followed and further a statement about the refolding pathway is possible. As the imino protons of the inner tetrad keep protected for HD exchange during the entire refolding process, only local rearrangements seem involved in the topological conversion.

The same transformation was observed in the presence of acetonitrile and PEG. Hence, the transition happened owing to a local dehydration effect that was persuaded by the incorporation of 8C3O into the solvent sphere of G4. This hydrophobicity in the vicinity of G4 triggered the formation of the parallel G4 form. Further, it proves that PEG‐induced structural changes can't be attributed to the molecular crowding but to the described dehydration effect. Thus, G4 structures determined with PEG might not represent the physiological relevant form.

## 3 Other G4‐interactive Carbazole Ligands

Apart from the above‐mentioned carbazole ligands, several other carbazole derivatives were reported and many of them emerged as promising candidates for G4 visualization^[40]^ and development of effective drug candidates for therapeutic purposes.^[41]^ Most of these ligands are substituted with different lateral chains to improve multiple properties, such as enhanced affinity for G4s, cellular uptake and solubility as well as efficient detection of intracellular G4 structures. However, their G4 binding capabilities were established by different means of biophysical studies other than detailed NMR analysis.

### 3.1 Fluorescent probes

**Figure 11:**
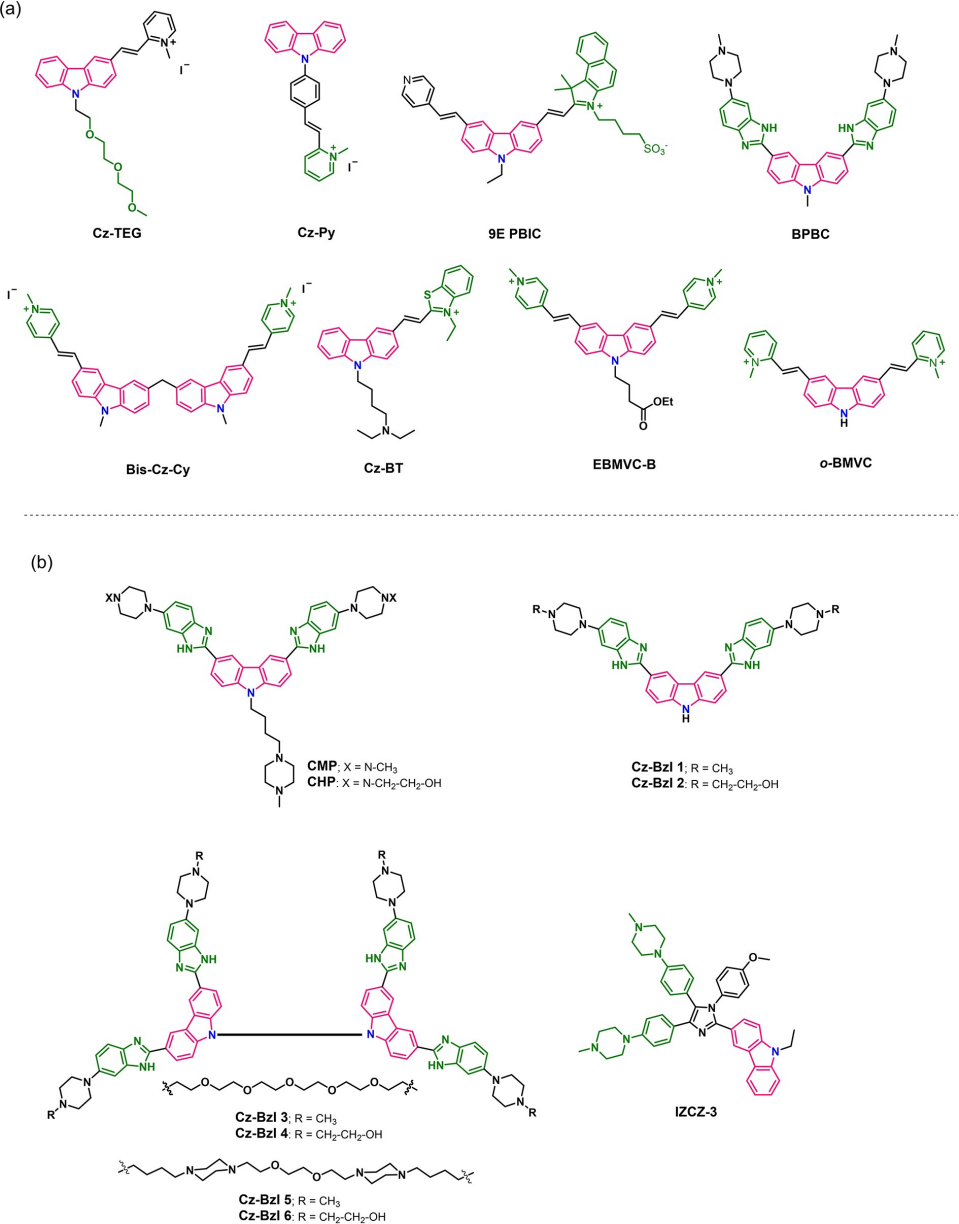
Chemical structures of other G4‐interactive carbazole ligands: the carbazole derived (a) fluorescent probes and (b) anti‐cancer agents.

The fused heteroaromatic nitrogen containing ring system of carbazole is an effective electron donating optical chromophore.^[42]^ The large π‐conjugated scaffold also displayed good Stokes shifts and high quantum yields owing to intramolecular charge transfer (ICT) along with excellent biocompatibility and stability. Thus, carbazoles are widely used as fluorescent turn on probes for sensitive and efficient monitoring of cellular G4s. A number of carbazole based fluorescent ligands with superior selectivity over the duplex DNA were reported (Figure 11a).

The triethylene glycol conjugated carbazole derivative **Cz**‐**TEG** ^[40a]^ and the groove binder cationic pyridinium containing analogue **Cz**‐**Py** ^[40b]^ exhibit superior G4 selectivity and low cytotoxicity that are useful for G4‐DNA sensing (Figure 11a). A benzindole substituted carbazole cyanine dye **9E PBIC** was reported as a selective fluorescent probe for parallel *c‐MYC* G4 DNA.^[40c]^ The dye selectively discriminates parallel *c‐MYC* 2345 G4 over other parallel, antiparallel and hybrid G4 and ss/ds DNAs by showing a 100‐fold fluorescence enhancement. Another light up probe, **BPBC** composed of benzimidazole and carbazole moieties was reported for the selective recognition of parallel G4s.^[40d]^ For visualization of cellular G4s, a two‐photon fluorescent probe **Cz‐BT** ^[40e]^ was synthesized by conjugating a benzothiazole moiety with the carbazole core with a diethyl amine side chain at the 9^th^ position. As the probe requires higher wavelength excitation (>700 nm), it could easily avoid the interference of autofluorescence within cellular environment. Later, a bis‐carbazolyl methane‐based cyanine derivative (Bis‐Cz‐Cy) was developed for two‐photon excited fluorescence (TPEF) microscopy imaging of G4s within cellular environment.^[40f]^ The probe shows excitation maxima at 820 nm and thus is practically more effective for non‐invasive live cell imaging due to deep penetration, low photobleaching and high spatial resolution. Another two‐photon excited ligand **EBMVC‐B** was employed with a modified G‐rich oligonucleotide (5'‐TGAG_3_AG_4_‐3'‐3'‐T‐5') to develop an intermolecular G4‐ligand complex that acts as a fluorescent sensor of blood potassium levels (Figure 11a).^[40g]^ The G‐rich oligonucleotide contains an inverted thymine nucleotide whose 3’‐terminus was connected to the 3’‐terminus of the upstream nucleotide blocking nuclease activity in biological fluids. The oligonucleotide folds into an intermolecular G4 specifically in the presence of K^+^ ions and subsequently interacts with the G4‐binder **EBMVC‐B** producing fluorescence turn on signals in response to K^+^ ions. Interestingly, the G4‐**EBMVC‐B** complex selectively detects K^+^ ions with no interference from other competitive ions like Na^+^, Li+, Ca^2+^, Mg^2+^, NH^4+^, Zn^2+^, Cu^2+^ etc. under physiological condition. The fluorescent carbazole iodide derivative BMVC exhibits high sensitivity for quadruplexes with *anti‐anti‐anti‐anti* and *anti‐anti‐syn‐syn* arrangements (anti‐parallel topology) while showing weak fluorescence responses for duplexes and quadruplexes with *anti‐syn‐anti‐syn* arrangement.^[40h]^ Further, another **BMVC** analogue containing two *ortho*‐pyridinium groups instead of *para‐*pyridinium, ***o‐*** **BMVC** has been developed showing a large contrast in fluorescence decay time, binding affinity, and fluorescent intensity between G4 structures.^[40i]^ Owing to its longer fluorescence decay times, the ligand is able to specifically visualize the location of G4 foci in living cells. By staining several cancer cells and normal cells with ***o*** **‐BMVC**, it could be demonstrated that significantly more G4 foci are present in cancer cells. In further studies with patient cells, ***o*** **‐BMVC** appears applicable as biosensor for human head and neck cancer.^[40j]^

### 3.2 Anticancer agents

Several derivatives of carbazoles have been explored as effective G4 targeting anticancer agents (Figure 11b).^[41]^ The group of Muniyappa and Bhattacharya introduced carbazole‐benzimidazole conjugates (**CMP** and **CHP**) for selective inhibition of telomerase activity.^[41a]^ Both these conjugates demonstrated fluorescence light‐up in the presence of *Hum21* telomeric G4 DNA and structural inversion from the K^+^‐stabilized hybrid G4 structure into a stable, telomeric parallel G4 DNA while showing distinct induced circular dichroism (ICD) signal with Na^+^ stabilized anti‐parallel *Hum21*. The bis‐benzimidazole ligands could inhibit telomerase function and induce cytotoxicity in telomerase positive cancer cells by selectively penetrating the nucleus of cancer cell lines over the normal telomerase negative primary cells. Inspired from these findings the group again developed six new carbazole based benzimidazoles for selective inhibition of telomerase activity (Figure 11b).^[41b]^ Among these series of carbazoles, the dimeric bis‐benzimidazoles with hydroxyethyl substituted piperazines (**Cz**‐**BzI 5**–**6**) demonstrated significantly high binding and stabilization capability for telomeric G4 DNA and induced cancer cell specific apoptosis. In a subsequent study, the group synthesized a series of other carbazole based benzimidazoles for targeting G4 structures in oncogene promoter regions.^[41c]^ All the ligands have been found to stabilize the G4 DNA of *c‐MYC, c‐KIT1, c‐KIT2, VEGF* and *BCL2* gene promoters and repress the expression of the oncogenes in cancer cells but they fail to discriminate among diverse G4 topologies.

Chen, Huang and Tan group developed a four‐leaf clover‐like ligand **IZCZ‐3** for specific stabilization of parallel topology of promoter *c‐MYC* G4 (Figure 11b).^[41d]^ The ligand was synthesized by conjugating a carbazole moiety with a triaryl‐substituted imidazole in a one‐pot condensation reaction. The ligand **IZCZ‐3** imparted high stability to *c‐MYC* G4 and showed little effect on other telomeric and promoter quadruplexes *HRAS, c‐KIT1, BCL2*, *KRAS, RET* and *PDGFA* as well as i‐motif, triplex, duplex and hairpin DNA. Molecular docking studies revealed that **IZCZ‐3** stacked perfectly on the terminal G‐tetrad and the central imidazole moiety was found to be located onto the ion channel of the *c‐MYC* G4. The conjugate efficiently inhibits *c‐MYC* transcription via binding and stabilizing the promoter *c‐MYC* G4 in cells leading to cell cycle arrest at G0/G1 phase and apoptosis. It also inhibits tumor growth in a human cervical squamous cancer xenograft.

## 4 Concluding Remarks

We presented here a large variety of carbazole ligands with respect to their possible binding modes and promising therapeutic and sensing applications. Most of them have been shown to suppress *c‐MYC* expression and induce apoptosis in cancer cells. **BMVC** is a promising anti‐cancer drug candidate which binds in low nM range, but it lacks selectivity for *c‐MYC* over other G4s.^[43]^ However, the reported solution NMR structure of the **BMVC**‐*c‐MYC22* complex can be used for targeted ligand design. The carbazole ligand **BTC f** augments the minor conformation of *c‐MYC* and preferentially binds to *c‐MYC* over *c‐KIT*. Therefore, ligands with conformational selection binding mode are probably good candidates for promoter selective G4 binding. **BMVC‐8C3O** even induces a change of the G4 into a parallel topology and could elucidate the PEG‐induced G4 structural changes as well as give insights into the pathway of folding transition from hybrid to parallel G4. Moreover, the large π‐conjugated system makes the carbazole ligands suitable as fluorescent probes for live cell imaging and potential anticancer agents. Altogether, the carbazole moiety can serve as an excellent core for designing DNA G4 ligands in the fields of nanotechnology, biomedical technology and anti‐cancer therapy.

## Conflict of interest

The authors declare no conflict of interest.

## Biographical Information

*Prof. Jyotirmayee Dash received her Ph.D. in Organic Chemistry from IIT Kanpur, India under the guidance of Professor F. A. Khan. She was awarded an Alexander von Humboldt fellowship with Professor H.‐U. Reissig, at the Free University of Berlin, and a postdoctoral fellowship with Professor J. Cossy, at ESPCI Paris. She was also a recipient of Marie Curie fellowship with Professor S. Balasubramanian, at the University of Cambridge, UK. In 2009, she joined IISER‐Kolkata as an Assistant Professor and she is working as a Professor at IACS‐Kolkata. She has been awarded the DST‐Swarnajayanti, DBT Wellcome Trust Fellowships and Shanti Swarup Bhatnagar Prize. Her research focuses on targeting the structure and function of of nucleic acid structures for therapeutic applications*.

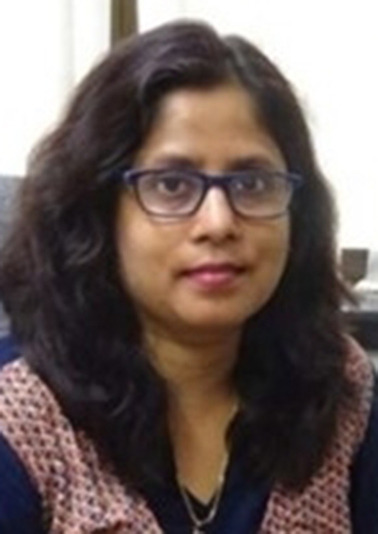

## Biographical Information

*Prof. Harald Schwalbe studied chemistry at the University of Frankfurt and obtained his PhD in 1993 with Christian Griesinger. He was postdoctoral fellow with Chris Dobson in Oxford from 1993*–*1995. After work on his habilitation till 1999, he became Assistant Professor at MIT from 1999*–*2001. In 2002, he accepted the offer to become full Professor at Goethe University in Frankfurt. His research focuses on NMR spectroscopy of protein, RNA, and DNA molecules with particular interest on their dynamics and complexes*.

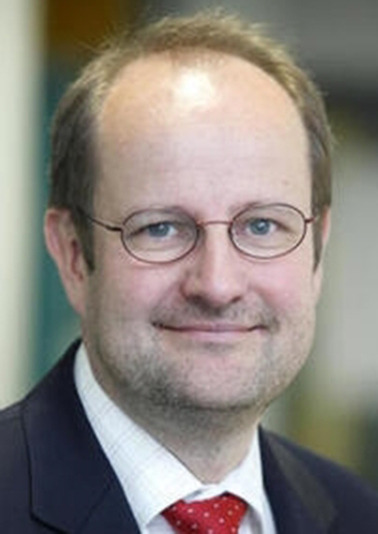

## References

[1a] H. Han , L. H. Hurley , Trends Pharmacol. Sci. 2000, 21, 136–142; 10740289 10.1016/s0165-6147(00)01457-7

[1b] S. Amrane , A. Kerkour , A. Bedrat , B. Vialet , M. L. Andreola , J. L. Mergny , J. Am. Chem. Soc. 2014, 136, 5249–5252; 24649937 10.1021/ja501500c

[1c] J. Spiegel , S. Adhikari , S. Balasubramanian , Trends Chem. 2020, 2, 123–136; 32923997 10.1016/j.trechm.2019.07.002 PMC7472594

[1d] R. Haensel-Hertsch , A. Simeone , A. Shea , W. W. I. Hui , K. G. Zyner , G. Marsico , O. M. Rueda , A. Bruna , A. Martin , X. Zhang , S. Adhikari , D. Tannahill , C. Caldas , S. Balasubramanian , Nat. Genet. 2020, 52, 878–883; 32747825 10.1038/s41588-020-0672-8

[1e] S. Sarkar , B. A. Armitage , ACS Infect. Dis. 2021, 6, 1445–1456; 10.1021/acsinfecdis.0c00793 33886274

[1f] J. Carvalho , J. L. Mergny , G. F. Salgado , J. A. Queiroz , C. Cruz , Trends Mol. Med. 2020, 26, 848–861; 32467069 10.1016/j.molmed.2020.05.002

[1g] E. Ruggiero , S. N. Richter , Nucleic Acids Res. 2018, 46, 3270–3283; 29554280 10.1093/nar/gky187 PMC5909458

[1h] L. M. Harris , C. J. Merrick , PLoS Pathog. 2015, 11, e1004562. 25654363 10.1371/journal.ppat.1004562 PMC4412290

[2a] D. Rhodes , H. J. Lipps , Nucleic Acids Res. 2015, 43, 8627–8637; 26350216 10.1093/nar/gkv862 PMC4605312

[2b] T. A. Brooks , S. Kendrick , L. Hurley , FEBS J. 2010, 277, 3459–3469; 20670278 10.1111/j.1742-4658.2010.07759.x PMC2971675

[2c] G. Biffi , D. Tannahill , J. McCafferty , S. Balasubramanian , Nat. Chem. 2013, 5, 182–186; 23422559 10.1038/nchem.1548 PMC3622242

[2d] A. Laguerre , K. Hukezalie , P. Winckler , F. Katranji , G. Chanteloup , M. Pirrotta , J. M. Perrier-Cornet , J. M. Wong , D. Monchaud , J. Am. Chem. Soc. 2015, 137, 8521–8525; 26056849 10.1021/jacs.5b03413

[2e] P. Prorok , M. Artufel , A. Aze , P. Coulombe , I. Peiffer , L. Lacroix , A. Guedin , J. L. Mergny , J. Damaschke , A. Schepers , C. Cayrou , M. P. Teulade-Fichou , B. Ballester , M. Mechali , Nat. Commun. 2019, 10, 3274; 31332171 10.1038/s41467-019-11104-0 PMC6646384

[2f] R. Haensel-Hertsch , D. Beraldi , S. V. Lensing , G. Marsico , K. Zyner , A. Parry , M. Di Antonio , J. Pike , H. Kimura , M. Narita , D. Tannahill , S. Balasubramanian , Nat. Genet. 2016, 48, 1267–1272; 27618450 10.1038/ng.3662

[2g] D. Rhodes , H. J. Lipps , Nucleic Acids Res. 2015, 43, 8627–8637. 26350216 10.1093/nar/gkv862 PMC4605312

[3a] S. Neidle , Nat. Chem. Rev. 2017, 1, 0041;

[3b] D. Varshney , J. Spiegel , K. Zyner , D. Tannahill , S. Balasubramanian , Nat. Rev. Mol. Cell Biol. 2020, 21, 459–474. 32313204 10.1038/s41580-020-0236-x PMC7115845

[4a] D. Monchaud , M. P. Teulade-Fichou , Org. Biomol. Chem. 2008, 6, 627–636; 18264563 10.1039/b714772b

[4b] S. Asamitsu , T. Bando , H. Sugiyama , Chem. Eur. J. 2019, 25, 417–430; 30051593 10.1002/chem.201802691

[4c] S. Neidle , J. Med. Chem. 2016, 59, 5987–6011; 26840940 10.1021/acs.jmedchem.5b01835

[4d] P. Saha , D. Panda , J. Dash , Chem. Commun. 2019, 55, 731–750; 10.1039/c8cc07107a 30489575

[4e] V. Pirota , M. Stasi , A. Benassi , F. Doria , in: Quadruplex Nucleic Acids As Targets For Medicinal Chemistry, (Ed: S. Neidle ) Academic Press, Cambridge, 2020, p. 163;

[4f] M. P. O′Hagan , J. C. Morales , M. C. Galan , Eur. J. Org. Chem. 2019, 31, 4995–5017;

[4g] J. Dash , P. Saha , K. Fatma , in: Quadruplex Nucleic Acids As Targets For Medicinal Chemistry, (Ed: S. Neidle ) Academic Press, Cambridge, 2020, p. 287;

[4h] D. Sen , W. Gilbert , Nature 1988, 334, 364–366. 3393228 10.1038/334364a0

[5a] Y. Wang , D. J. Patel , Structure 1993, 1, 263–282; 8081740 10.1016/0969-2126(93)90015-9

[5b] G. N. Parkinson , M. P. Lee , S. Neidle , Nature 2002, 417, 876–880; 12050675 10.1038/nature755

[5c] K. N. Luu , A. T. Phan , V. Kuryavyi , L. Lacroix , D. J. Patel , J. Am. Chem. Soc. 2006, 128, 9963–9970; 16866556 10.1021/ja062791w PMC4692383

[5d] S. Burge , G. N. Parkinson , P. Hazel , A. K. Todd , S. Neidle , Nucleic Acids Res. 2006, 34, 5402–5415; 17012276 10.1093/nar/gkl655 PMC1636468

[5e] D. J. Patel , A. T. Phan , V. Kuryavyi , Nucleic Acids Res. 2007, 35, 7429–7455; 17913750 10.1093/nar/gkm711 PMC2190718

[5f] S. Neidle , Curr. Opin. Struct. Biol. 2009, 19, 239–250; 19487118 10.1016/j.sbi.2009.04.001

[5g] M. Adrian , B. Heddi , A. T. Phan , Methods 2012, 57, 11–24. 22633887 10.1016/j.ymeth.2012.05.003

[6a] A. W. Schmidt , K. R. Reddy , H. J. Knolker , Chem. Rev. 2012, 112, 3193–3328; 22480243 10.1021/cr200447s

[6b] H. J. Knölker , K. R. Reddy , Chem. Rev. 2002, 102, 4303–4427. 10.1021/cr020059j 12428991

[7a] A. Gluszynska , Eur. J. Med. Chem. 2015, 94, 405–426; 25794500 10.1016/j.ejmech.2015.02.059

[7b] M. Bashir , A. Bano , A. S. Ijaz , B. A. Chaudhary , Molecules 2015, 20, 13496–13517. 26213906 10.3390/molecules200813496 PMC6332089

[8a] D. P. Chakraborty , B. K. Barman , P. K. Bose , Tetrahedron 1965, 21, 681–685;

[8b] Y. Nalli , V. Khajuria , S. Gupta , P. Arora , S. Riyaz-Ul-Hassan , Z. Ahmed , A. Ali , Org. Biomol. Chem. 2016, 14, 3322–3332. 26947457 10.1039/c6ob00267f

[9a] H. J. Knölker , M. Wolpert , Tetrahedron Lett. 1997, 38, 533–536;

[9b] F. Liger , F. Popowycz , T. Besson , L. Picot , C. M. Galmarini , B. Joseph , Bioorg. Med. Chem. 2007, 15, 5615–5619. 17548198 10.1016/j.bmc.2007.05.033

[10] K. Sakano , K. Ishimaru , S. Nakamura , J. Antibiot. 1980, 33, 683–689. 10.7164/antibiotics.33.683 7410212

[11] N. Kotola , K. S. Ya , K. Furihata , Y. Hayakawa , H. Seto , J. Antibiot. 1997, 50, 770–772. 10.7164/antibiotics.50.770 9360623

[12] A. Furusaki , N. Hashiba , T. Matsumoto , A. Hirano , Y. Iwai , S. J. Omura , J. Chem. Soc. Chem. Commun. 1978, 18, 800–801.

[13] T. Martin , C. J. Moody , Tetrahedron Lett. 1985, 26, 5841–5842.

[14a] R. B. Woodward , G. A. Iacobucci , I. A. Hochstein , J. Am. Chem. Soc. 1959, 81, 4434–4435;

[14b] V. M. Dan , T. S. Varghese , G. Viswanathan , S. Baby , Curr. Cancer Drug Targets 2020, 20, 33–46. 31560288 10.2174/1568009619666190927150131

[15] E. Wenkert , K. G. Dave , J. Am. Chem. Soc. 1962, 84, 94–97.

[16] G. W. Gribble , in: The Alkaloids. Chemistry and Pharmacology, (Ed: A. Brossi ) Academic Press, New York, 1990, p. 239.

[17] S. Issa , A. Prandina , N. Bedel , P. Rongved , S. Yous , M. Le Borgne , Z. Bouaziz , J. Enzyme Inhib. Med. Chem. 2019, 34, 1321–1346. 31328585 10.1080/14756366.2019.1640692 PMC6691762

[18a] I. Bessi , J. Wirmer-Bartoschek , J. Dash , H. Schwalbe , in: Modern Magnetic Resonance, (Ed: G. A. Webb ) Springer International Publishing AG, Cham, 2017, p. 2189;

[18b] C. Lin , D. Yang , Methods Mol. Biol. 2017, 1587, 171–196; 28324509 10.1007/978-1-4939-6892-3_17 PMC5516791

[18c] C. Lin , J. Dickerhoff , D. Yang , Methods Mol. Biol. 2019, 2035, 157–176. 31444749 10.1007/978-1-4939-9666-7_9 PMC7263470

[19] A. T. Phan , J. -L Mergny , Nucleic Acids Res. 2002, 30, 4618–4625. 12409451 10.1093/nar/gkf597 PMC135813

[20] O. Binas , I. Bessi , H. Schwalbe , ChemBioChem 2020, 21, 1656–1663. 31943589 10.1002/cbic.201900696 PMC7318348

[21a] L. Y. Liu , W. Liu , K. N. Wang , B. C. Zhu , X. Y. Xia , L. N. Ji , Z. W. Mao , Angew. Chem. Int. Ed. 2020, 59, 9719–9726; 10.1002/anie.202002422 32173994

[21b] L. Martino , A. Virno , B. Pagano , A. Virgilio , S. Di Micco , A. Galeone , C. Giancola , G. Bifulco , L. Mayol , A. Randazzo , J. Am. Chem. Soc. 2007, 129, 16048–16056; 18052170 10.1021/ja075710k

[21c] S. Cosconati , L. Marinelli , R. Trotta , A. Virno , S. De Tito , R. Romagnoli , B. Pagano , V. Limongelli , C. Giancola , P. G. Baraldi , L. Mayol , E. Novellino , A. Randazzo , J. Am. Chem. Soc. 2010, 132, 6425–6433; 20394365 10.1021/ja1003872

[21d] E. Gavathiotis , R. A. Heald , M. F. Stevens , M. S. Searle , J. Mol. Biol. 2003, 334, 25–36; 14596797 10.1016/j.jmb.2003.09.018

[21e] J. Wirmer-Bartoschek , L. E. Bendel , H. R. Jonker , J. T. Grün , F. Papi , C. Bazzicalupi , L. Messori , P. Gratteri , H. Schwalbe , Angew. Chem. Int. Ed. 2017, 56, 7102–7106; 10.1002/anie.201702135 28524432

[21f] W. Liu , Y.-F. Zhong , L.-Y. Liu , C.-T. Shen , W. Zeng , F. Wang , D. Yang , Z.-W. Mao , Nat. Commun. 2018, 9, 1–11; 30158518 10.1038/s41467-018-05810-4 PMC6115404

[21g] A. T. Phan , V. Kuryavyi , H. Y. Gaw , D. J. Patel , Nat. Chem. Biol. 2005, 1, 167–173; 16408022 10.1038/nchembio723 PMC4690526

[21h] F. Wang , C. Wang , Y. Liu , W. Lan , H. Han , R. Wang , S. Huang , C. Cao , Chem. Commun. 2020, 56, 2099–2102; 10.1039/d0cc00221f 32025680

[21i] C. Lin , G. Wu , K. Wang , B. Onel , S. Sakai , Y. Shao , D. Yang , Angew. Chem. Int. Ed. 2018, 57, 10888–10893; 10.1002/anie.201804667 PMC6192034 29888501

[21j] J. Dai , M. Carver , L. H. Hurley , D. Yang , J. Am. Chem. Soc. 2011, 133, 17673–17680; 21967482 10.1021/ja205646q PMC3207019

[21k] W. J. Chung , B. Heddi , M. Tera , K. Iida , K. Nagasawa , A. T. Phan , J. Am. Chem. Soc. 2013, 135, 13495–13501; 23909929 10.1021/ja405843r

[21l] T. Wilson , P. J. Costa , V. Felix , M. P. Williamson , J. A. Thomas , J. Med. Chem. 2013, 56, 8674–8683; 24088028 10.1021/jm401119b PMC3835060

[21m] W. J. Chung , B. Heddi , F. Hamon , M. P. Teulade-Fichou , A. T. Phan , Angew. Chem. Int. Ed. 2014, 53, 999–1002; 10.1002/anie.201308063 24356977

[21n] A. Tawani , A. Kumar , Sci. Rep. 2015, 5, 17574; 26627543 10.1038/srep17574 PMC4667226

[21o] C. Hounsou , L. Guittat , D. Monchaud , M. Jourdan , N. Saettel , J. L. Mergny , M. P. Teulade-Fichou , ChemMedChem 2007, 2, 655–666; 17385760 10.1002/cmdc.200600286

[21p] A. Kotar , B. Wang , A. Shivalingam , J. Gonzalez-Garcia , R. Vilar , J. Plavec , Angew. Chem. Int. Ed. 2016, 55, 12508–12511; 10.1002/anie.201606877 27577037

[21q] D. R. Calabrese , X. Chen , E. C. Leon , S. M. Gaikwad , Z. Phyo , W. M. Hewitt , S. Alden , T. A. Hilimire , F. He , A. M. Michalowski , J. K. Simmons , L. B. Saunders , S. Zhang , D. Connors , K. J. Walters , B. A. Mock , J. S. Schneekloth Jr. , Nat. Commun. 2018, 9, 4229; 30315240 10.1038/s41467-018-06315-w PMC6185959

[21r] R. Barthwal , S. Raje , K. Pandav , J. Biomol. Struct. Dyn. 2021, 39, 795–815; 32070245 10.1080/07391102.2020.1730969

[21s] I. Gomez-Pinto , E. Vengut-Climent , R. Lucas , A. Avino , R. Eritja , C. Gonzalez , J. C. Morales , Chemistry 2013, 19, 1920–1927. 23315826 10.1002/chem.201203902

[22a] A. Chauhan , R. Paul , M. Debnath , I. Bessi , S. Mandal , H. Schwalbe , J. Dash , J. Med. Chem. 2016, 59, 7275–7281; 27442915 10.1021/acs.jmedchem.6b00328

[22b] J. Carvalho , P. Nottelet , J. L. Mergny , J. A. Queiroz , G. F. Salgado , C. Cruz , Biochimie 2017, 135, 186–195. 28232078 10.1016/j.biochi.2017.02.005

[23] G. G. Hammes , Y.-C. Chang , T. G. Oas , Proc. Nat. Acad. Sci. 2009, 106, 13737–13741. 19666553 10.1073/pnas.0907195106 PMC2728963

[24a] D. Panda , P. Saha , T. Das , J. Dash , Nat. Commun. 2017, 8, 16103; 28706243 10.1038/ncomms16103 PMC5519986

[24b] P. Saha , D. Panda , D. Müller , A. Maity , H. Schwalbe , J. Dash , Chem. Sci. 2020, 11, 2058–2067. 32180928 10.1039/d0sc00514b PMC7047845

[25] X.-F. Zhang , H.-J. Zhang , J.-F. Xiang , Q. Li , Q.-F. Yang , Q. Shang , Y.-X. Zhang , Y.-L. Tang , J. Mol. Struct. 2010, 982, 133–138.

[26] A. Ou , A. Guédin , B. W. Skelton , S. Amrane , C. W. Evans , M. Norret , K. S. Iyer , J.-L. Mergny , N. M. Smith , Chem. Commun. 2018, 54, 9647–9650. 10.1039/c8cc03945c 30101241

[27] S. Jana , D. Panda , P. Saha , G. D. Pantos̨ , J. Dash , J. Med. Chem. 2019, 62, 762–773. 30525583 10.1021/acs.jmedchem.8b01459

[28] T. Das , D. Panda , P. Saha , J. Dash , Bioconjugate Chem. 2018, 29, 2636–2645. 10.1021/acs.bioconjchem.8b00338 29956928

[29] A. Ambrus , D. Chen , J. Dai , R. A. Jones , D. Yang , Biochemistry 2005, 44, 2048–2058. 15697230 10.1021/bi048242p

[30] W. Liu , C. Lin , G. Wu , J. Dai , T. C. Chang , D. Yang , Nucleic Acids Res. 2019, 47, 11931–11942. 31740959 10.1093/nar/gkz1015 PMC7145684

[31] Y.-L. Tsai , Z.-F. Wang , W.-W. Chen , T.-C. Chang , Nucleic Acids Res. 2011, 39, e114–e114. 21715373 10.1093/nar/gkr499 PMC3177220

[32] K. N. Luu , A. T. Phan , V. Kuryavyi , L. Lacroix , D. J. Patel , J. Am. Chem. Soc. 2006, 128, 9963–9970. 16866556 10.1021/ja062791w PMC4692383

[33] L. Hu , K. W. Lim , S. Bouaziz , A. T. Phan , J. Am. Chem. Soc. 2009, 131, 16824–16831. 19874015 10.1021/ja905611c

[34] A. T. Phan , Y. S. Modi , D. J. Patel , J. Am. Chem. Soc. 2004, 126, 8710–8716. 15250723 10.1021/ja048805k PMC4692381

[35] J. Dai , D. Chen , R. A. Jones , L. H. Hurley , D. Yang , Nucleic Acids Res. 2006, 34, 5133–5144. 16998187 10.1093/nar/gkl610 PMC1636422

[36] M. Debnath , S. Ghosh , D. Panda , I. Bessi , H. Schwalbe , K. Bhattacharyya , J. Dash , Chem. Sci. 2016, 7, 3279–3285. 29997820 10.1039/c6sc00057f PMC6006475

[37] D. Panda , M. Debnath , S. Mandal , I. Bessi , H. Schwalbe , J. Dash , Sci. Rep. 2015, 5, 13183. 26286633 10.1038/srep13183 PMC4541407

[38] Y. Gu , D. Lin , Y. Tang , X. Fei , C. Wang , B. Zhang , J. Zhou , Spectrochim. Acta Part A 2018, 191, 180–188. 10.1016/j.saa.2017.10.012 29032342

[39a] Z.-F. Wang , M.-H. Li , W.-W. Chen , S.-T. D. Hsu , T.-C. Chang , Nucleic Acids Res. 2016, 44, 3958–3968; 26975658 10.1093/nar/gkw145 PMC4856992

[39b] Z.-F. Wang , T.-C. Chang , Nucleic Acids Res. 2012, 40, 8711–8720. 22735707 10.1093/nar/gks578 PMC3458535

[40a] Q. Q. Yu , M. Q. Wang , Bioorg. Med. Chem. 2020, 28, 115641; 32773092 10.1016/j.bmc.2020.115641

[40b] M. Q. Wang , G. Y. Ren , S. Zhao , G. C. Lian , T. T. Chen , Y. Ci , H. Y. Li , Spectrochim. Acta A. 2018, 199, 441–447; 10.1016/j.saa.2018.03.083 29649680

[40c] D. Lin , X. Fei , Y. Gu , C. Wang , Y. Tang , R. Li , J. Zhou , Analyst 2015, 140, 5772–5780; 26176020 10.1039/c5an00866b

[40d] B. Jin , X. Zhang , W. Zheng , X. Liu , C. Qi , F. Wang , D. Shangguan , Anal. Chem. 2014, 86, 943–952; 24354276 10.1021/ac403676x

[40e] F. Gao , S. Cao , W. Sun , S. Long , J. Fan , X. Peng , Dyes Pigm. 2019, 171, 107749;

[40f] Y. C. Zheng , M. L. Zheng , S. Chen , Z. S. Zhao , X. M. Duan , J. Mater. Chem. B 2014, 2, 2301–2310; 32261718 10.1039/c3tb21860k

[40g] L. Yang , Z. Qing , C. Liu , Q. Tang , J. Li , S. Yang , J. Zheng , R. Yang , W. Tan , Anal. Chem. 2016, 88, 9285–9292; 27558922 10.1021/acs.analchem.6b02667

[40h] C. C. Chang , J. Y. Wu , C. W. Chien , W. S. Wu , H. Liu , C. C. Kang , L. J. Yu , T. C. Chang , Anal. Chem. 2003, 75, 6177–6183; 14615998 10.1021/ac034789i

[40i] T. Y. Tseng , C. H. Chien , J. F. Chu , W. C. Huang , M. Y. Lin , C. C. Chang , T. C. Chang , J. Biomed. Opt. 2013, 18, 101309; 23839279 10.1117/1.JBO.18.10.101309

[40j] T. Y. Tseng , W. W. Chen , I. T. Chu , C. L. Wang , C. C. Chang , M. C. Lin , P. J. Lou , T. C. Chang , Sci. Rep. 2018, 8, 16082. 30382130 10.1038/s41598-018-34378-8 PMC6208391

[41a] B. Maji , K. Kumar , M. Kaulage , K. Muniyappa , S. Bhattacharya , J. Med. Chem. 2014, 57, 6973–6988; 25062468 10.1021/jm500427n

[41b] B. Maji , K. Kumar , K. Muniyappa , S. Bhattacharya , Org. Biomol. Chem. 2015, 13, 8335–8348; 26149178 10.1039/c5ob00675a

[41c] M. H. Kaulage , B. Maji , S. Pasadi , A. Ali , S. Bhattacharya , K. Muniyappa , Eur. J. Med. Chem. 2018, 148, 178–194; 29459277 10.1016/j.ejmech.2018.01.091

[41d] M. H. Hu , Y. Q. Wang , Z. Y. Yu , L. N. Hu , T. M. Ou , S. B. Chen , Z. S. Huang , J. H. Tan , J. Med. Chem. 2018, 61, 2447–2459; 29474069 10.1021/acs.jmedchem.7b01697

[41e] Y. Wei , X. Zhang , L. Wang , Y. Liu , T. Bing , X. Liu , D. Shangguan , RSC Adv. 2015, 5, 75911–75917;

[41f] A. Gluszynska , B. Juskowiak , M. Kuta-Siejkowska , M. Hoffmann , S. Haider , Int. J. Biol. Macromol. 2018, 114, 479–490; 29581003 10.1016/j.ijbiomac.2018.03.135

[41g] J. Li , Q. Yang , L. Zhao , M. Xu , H. Zhang , Tetrahedron Lett. 2021, 70, 153004.

[42a] K. R. J. Thomas , J. T. Lin , Y. T. Tao , C. K. Ko , J. Am. Chem. Soc. 2001, 123, 9401–9411; 10.1021/ja010819s 11562223

[42b] K. Brunner , A. V. Dijken , H. Bomer , J. J. A. M. Bastlaansen , N. M. M. Kiggen , B. M. W. Langeveld , J. Am. Chem. Soc. 2004, 126, 6035–6042; 15137768 10.1021/ja049883a

[42c] H. X. Shao , X. P. Chen , Z. X. Wang , P. Lu , J. Lumin. 2007, 127, 349–354;

[42d] S. M. Song , D. Ju , J. F. Li , D. X. Li , Y. L. Wei , C. Dong , P. H. Lin , S. M. Shuang , Talanta 2009, 77, 1707–1714; 19159787 10.1016/j.talanta.2008.10.008

[42e] A. Casey , R. S. Ashraf , Z. P. Fei , M. Heene , Macromolecules 2014, 47, 2279–2288.

[43] G. Wu , D. Tillo , S. Ray , T. C. Chang , J. S. Schneekloth Jr. , C. Vinson , D. Yang , Molecules 2020, 25, 3465. 10.3390/molecules25153465 PMC7436161 32751510

